# Supplementary material for: Translocation of a single Arg9 peptide across a DOPC/DOPG(4:1) model membrane using the weighted ensemble method
Source: Sci Rep. 2023 Jan 20;13:1168. doi: 10.1038/s41598-023-28493-4 (PMC9860060; doi:10.1038/s41598-023-28493-4)
Supplement: Supplementary file 1 — Supplementary Information. [file 41598_2023_28493_MOESM1_ESM.pdf]

Supplementary Information

Translocation of a single Arg_9 peptide across a DOPC/DOPG(4:1) model membrane using the weighted ensemble method

Seungho Choe[1],[2]

[1] *Department of Energy Science & Engineering, DGIST, Daegu 42988, South Korea*

[2] *Energy Science & Engineering Research Center, DGIST, Daegu 42988, South Korea*

I. THE NUMBER OF HYDROGEN BONDS BETWEEN ARG_9 AND THE LIPIDS MOLECULES AND THE CONFORMATIONAL CHANGE OF ARG_9 DURING THE TRANSLOCATION

We counted the number of hydrogen bonds between Arg_9 and the lipid molecules during the WE simulations. Fig. S1 denotes the number of hydrogen bonds calculated in VMD [1]. The blue line is the number of the hydrogen bonds between Arg_9 and the lipids (including the phosphate group), while the green line is between Arg_9 and only the phosphate group. On the other hand, the red line denotes the hydrogen bonds between Arg_9 and water. Each point in the figure corresponds to an averaged value over 50 ps. The red line shows a rapid increase when Arg_9 reaches the bottom of the membrane. The figure shows that the average number of hydrogen bonds between Arg_9 and both the lipids and water was about 20. We suggest in the main text that the water flow and the orientation angle of Arg_9 can play a role in the translocation of Arg_9.

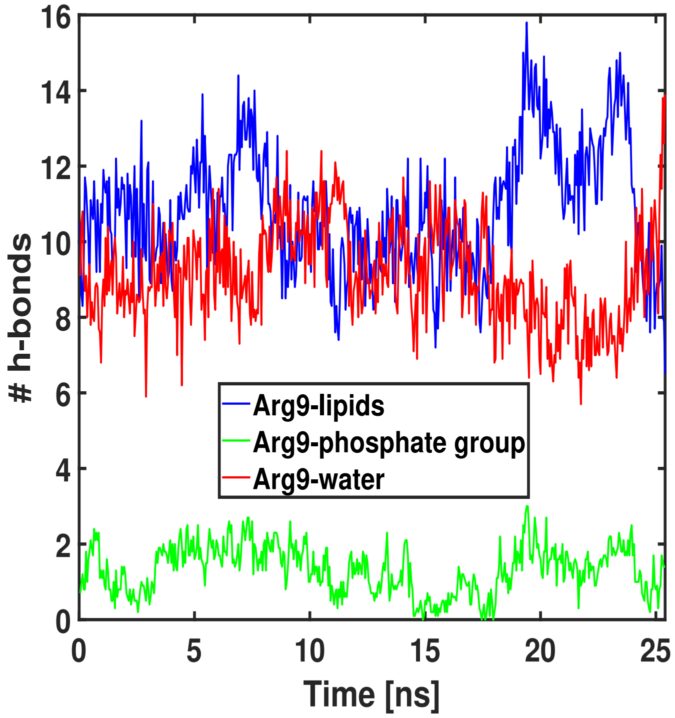

FIG. S1: The number of hydrogen bonds (blue: between Arg_9 and the lipids molecules (including the phosphate group), green: between Arg_9 and only the phosphate group, red: between Arg_9 and water) vs. the simulation time

Fig. S2 shows the conformational change of Arg_9s during the WE simulation calculated in the Timeline in VMD [1]. The third Arg_9 is the one that showed the translocation across the membrane, and most of the residues maintained “turn” conformation during the whole WE simulation. The other three Arg_9s showed the conformation of the random coil.

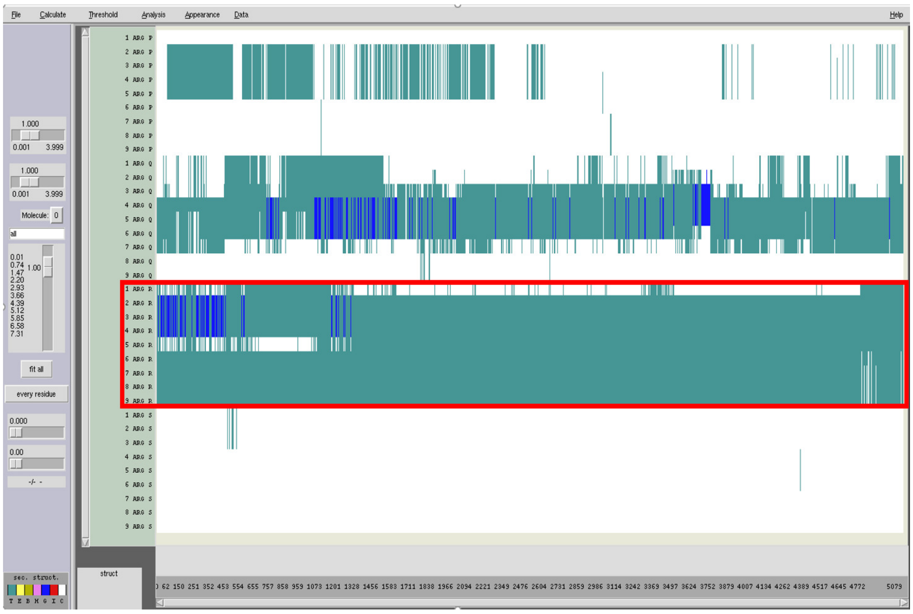

FIG. S2: The conformational change of Arg_9s during the WE simulation. We used the Timeline in VMD [1]. The third Arg_9 is the one that showed the translocation across the membrane

II. COMPARISON OF THE ORIENTATION ANGLE OF ARG$_9$ AT THE LAST PART OF THE TRANSLOCATION

In addition to the WE5 simulation, we performed two more simulations (WE5a and WE5b) to compare the orientation angle of Arg_9 during the last part of the translocation to the bottom of the membrane. We used the same system set up in the WE5 simulation for the WE5a and WE5b simulations. Therefore, they have the same boundary and bin size. Table S1 denotes a list of three WE simulations performed at the end of translocation, including the WE5 simulation in the main text. Fig. S3 shows the penetration depth vs. the orientation angle from the WE5, WE5a, and WE5b simulations. The figure shows a strong correlation between the penetration depth and the orientation angle. Arg_9 can penetrate

rapidly with a smaller orientation angle.

TABLE S1: A list of three WE simulations performed at the last part of the translocation

simulation no.	bin boundaries	bin size (the smallest)	# iterations
WE5	[−45.0 Å, −33.0 Å]	0.10 Å	196
WE5a	[−45.0 Å, −33.0 Å]	0.10 Å	315
WE5b	[−45.0 Å, −33.0 Å]	0.10 Å	390

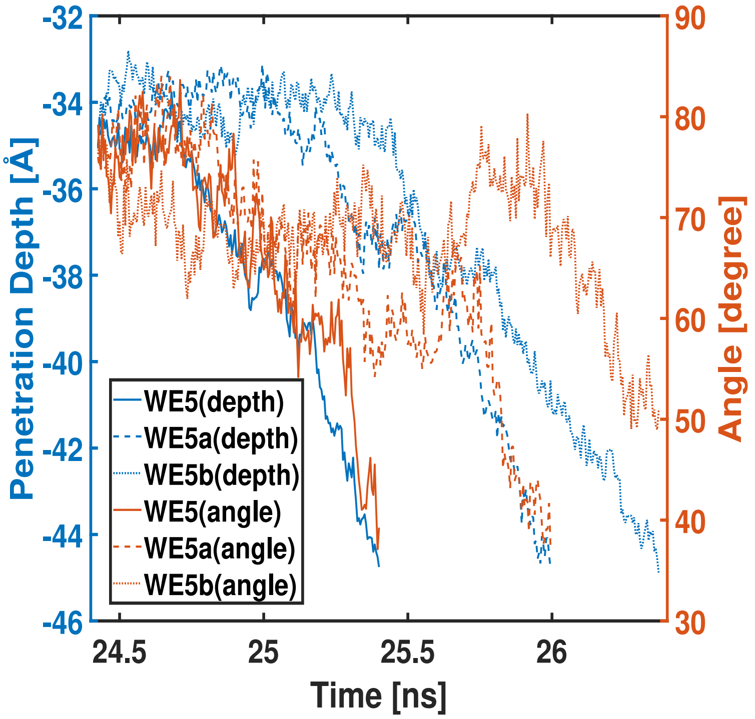

FIG. S3: The penetration depth and the orientation angle of Arg_9 from three different simulations: WE5, WE5a, and WE5b (the last part of the translocation) vs. the simulation time. The time was counted since the WE1 simulation started (see Table 1 in the main text).

III. AN ADDITIONAL CONVENTIONAL MD SIMULATION WITHOUT THE WE METHOD

After finishing the WE simulations (from the WE1 to WE5), we ran an additional conventional MD simulation for 1 μs to see conformational changes of Arg_9s and the lipid molecules. The simulation method was similar to the previous ones[2, 3]. Fig. S4 presents snapshots at a few different times during the simulation. During the simulation, Arg_9 moved back to the membrane's center with a few lipid molecules. The main text mentioned that six lipid molecules moved along with Arg_9 during the WE simulations. Four stayed in the lower leaflet until the end of the simulation, while two moved back to the upper leaflet when Arg_9 moved back to the center of the membrane. Since the simulation started, it took about 45 ns for two lipids to move back to the upper leaflet. The water flow and the movement of Arg_9 affect the phospholipid(PL) translocation. The water pore was closed after about 70 ns, as shown in Fig. S5. Fig. S5 presents the total number of water molecules translocated across the membrane during the first 100 ns simulation. Before closing the water pore ($\sim$ 70 ns), the upward water flux was 1.5 (water molecules/ns), while the downward water flux was 1.2. The upward water flux is slightly higher than the downward water flux due to the upward movement of Arg_9.

[1] W. Humphrey, A. Dalke, and K. Schulten, J. Molec. Graphics **14**, 33 (1996).

[2] S. Choe, AIP Advances **10**, 105103 (2020).

[3] S. Choe, Membranes **11**, 974 (2021).

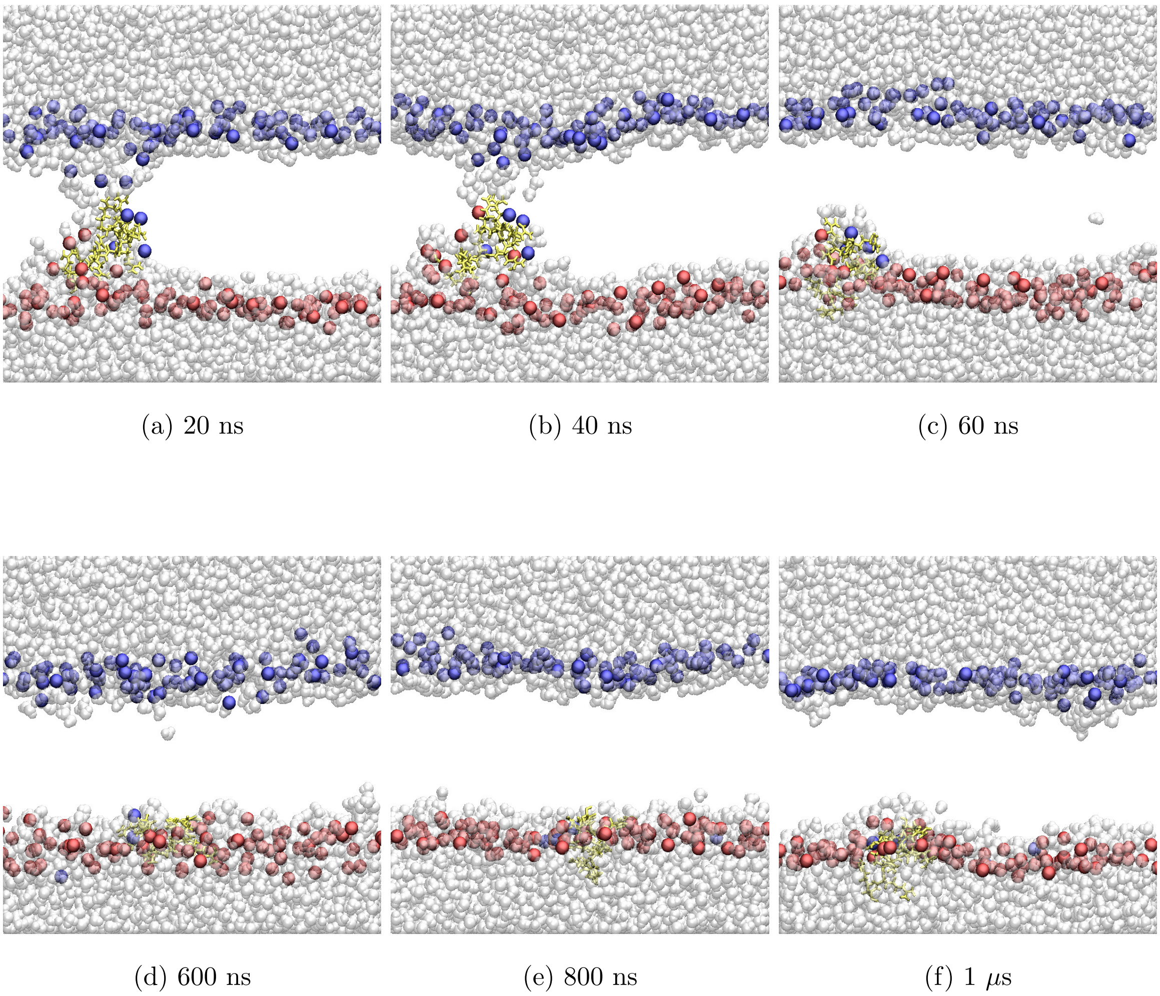

FIG. S4: Snapshots at (a) 20 ns, (b) 40 ns, (c) 60 ns, (d) 600 ns, (e) 800 ns, and (f) 1 μs during the additional conventional MD simulation (yellow: Arg_9, white spheres: water, blue & red : phosphorus atoms of the upper leaflet & lower leaflet, respectively).

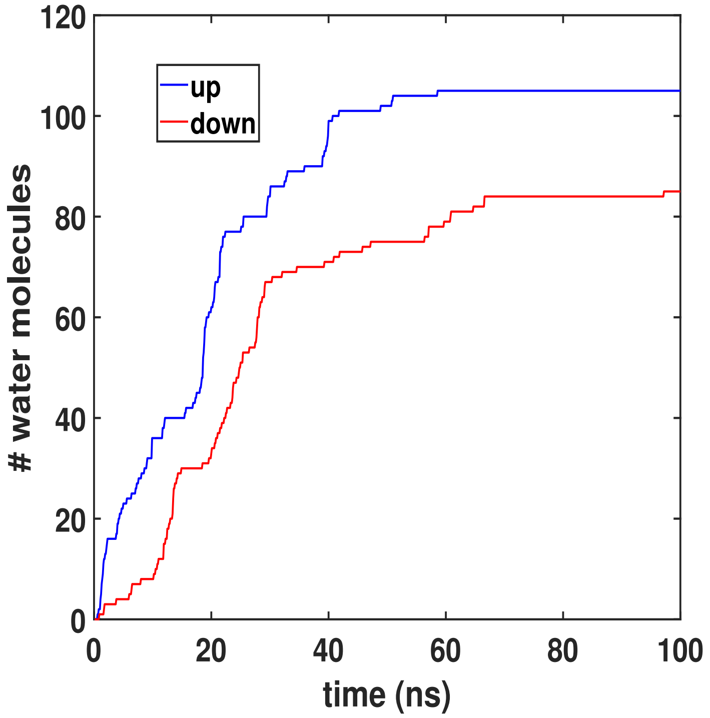

FIG. S5: The total accumulated number of water molecules translocated across the membrane (the moving-up(exiting) water & the moving-down(entering) water) vs. the simulation time
